# Improving the photovoltaic performance of perovskite solar cells with acetate

**DOI:** 10.1038/srep38670

**Published:** 2016-12-09

**Authors:** Qian Zhao, G. R. Li, Jian Song, Yulong Zhao, Yinghuai Qiang, X. P. Gao

**Affiliations:** 1 Institute of New Energy Material Chemistry, School of Materials Science and Engineering, National Institute of Advanced Materials, Nankai University, Tianjin 300350, China; 2 School of Materials Science and Engineering, China University of Mining and Technology, Xuzhou 221116, Jiangsu, China

## Abstract

In an all-solid-state perovskite solar cell, methylammonium lead halide film is in charge of generating photo-excited electrons, thus its quality can directly influence the final photovoltaic performance of the solar cell. This paper accentuates a very simple chemical approach to improving the quality of a perovskite film with a suitable amount of acetic acid. With introduction of acetate ions, a homogeneous, continual and hole-free perovskite film comprised of high-crystallinity grains is obtained. UV-visible spectra, steady-state and time-resolved photoluminescence (PL) spectra reveal that the obtained perovskite film under the optimized conditions shows a higher light absorption, more efficient electron transport, and faster electron extraction to the adjoining electron transport layer. The features result in the optimized perovskite film can provide an improved short-circuit current. The corresponding solar cells with a planar configuration achieves an improved power conversion efficiency of 13.80%, and the highest power conversion efficiency in the photovoltaic measurements is up to 14.71%. The results not only provide a simple approach to optimizing perovskite films but also present a novel angle of view on fabricating high-performance perovskite solar cells.

Organic−inorganic hybrid perovskite materials have undergone important developments for solar cells since they possess wide spectral absorption, long charge diffusion and ambipolar transport ability^1^,^2^,^3^,^4^. With methylammonium or formamidinium lead halide as the active layer for generating photo-excited electrons, the perovskite solar cells fabricated under certain conditions have achieved a comparable power-conversion efficiency (PCE) with commercial silicon solar cells^5^,^6^,^7^,^8^,^9^,^10^. At present, achieving high power-conversion efficiency and excellent stability is still the chief mission allocated to all researchers in developing perovskite solar cells.

The final performance of a perovskite solar cell is closely related to the quality of its perovskite film that is usually formed by a so-called one-step or two-step method combined with a solution or vapour deposition process^4^,^11^,^12^,^13^,^14^. An ideal perovskite film should be homogeneous, continual and composed of high-crystallinity large grains with few vacancies; moreover, it should have the relatively large contact area for hole-transport layer to form a compact interface. Some efforts have been made in order to obtain high-quality perovskite films, such as optimizing preparation conditions^14^,^15^,^16^, selecting starting materials^9^,^17^, and introducing modifying agents^18^,^19^. An ultra-smooth perovskite thin-film can be prepared by using lead acetate as lead source, while the grain size of perovskite crystals is smaller compared to that based on lead chloride^20^. When lead acetate is used, a so-called solvent-annealing method is helpful in increasing the perovskite grain size^21^. Large perovskite grains can also be formed from lead acetate mixed with lead chloride as lead source^22^. Enhanced reproducibility and low hysteresis for p-i-n perovskite solar cells can be achieved through the precursor containing lead acetate^23^. It is noted from the above works that lead acetate as lead source can be beneficial for obtaining high-quality perovskite films under the certain conditions. In addition, lead acetate has also been used to generate single crystal nanowires, nanorods, and nanoplates of perovskite through a dissolution-recrystallization pathway^24^. However, the real influence of acetate ions on the formation of perovskite films has not been investigated in detail yet. Actually, it has been found that some preferred generations of crystals and directional formation of films could be controlled by acetate ions^25^,^26^. More importantly, the research on the crystallization kinetics of perovskite reveals the elaborated isothermal transformations of perovskite films formed from different lead salts, and indicates that the activation energy of acetate system is low and coarsening of crystal occurs after the complete crystallization^27^. All the above works suggest that acetate ions play an important role in the formation process of perovskite film, and use of moderate acetate could have a beneficial effect on formation of a high-quality perovskite film.

In this paper, the effects of acetate ions on the morphology and structure, electrical properties, and photoelectrical performance of perovskite films are investigated. It is clearly shown that the introduction of acetate ions can improve the quality of perovskite films, influence its electrical properties, and enhance the final power conversion efficiency of the corresponding perovskite solar cells.

## Results

### Morphology and structure of perovskite films

**Figure 1:**
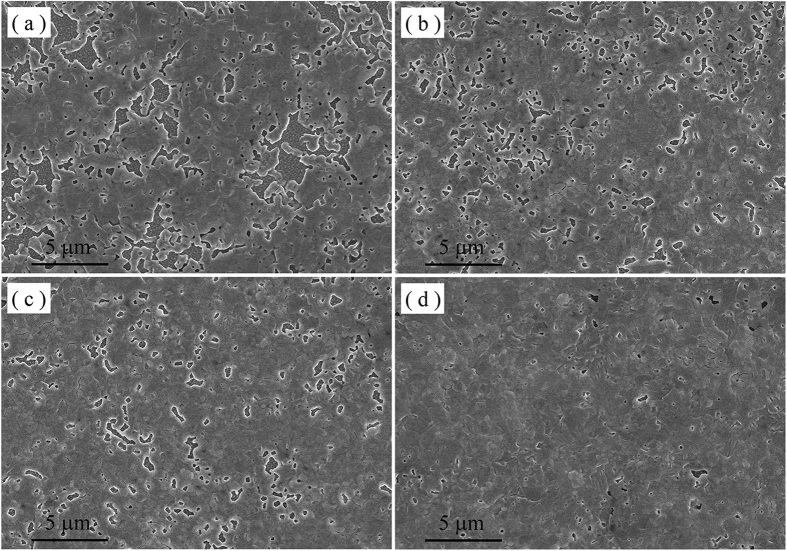
The morphology of films based on PbI_2_ by SEM. (**a**–**d**) The image of perovskite films deposited on a FTO/compact-TiO_2_ substrate from the precursor solution containing different amounts of HAc, 0 M, 0.25 M, 0.5 M and 1.0 M, respectively.

The morphologic changes of the perovskite films formed with different amounts of HAc can be directly observed by SEM. In the absence of HAc, the obtained perovskite film based on PbI_2_ as lead source is obviously discontinuous with a mass of irregular and micron-scale holes (Fig. 1a). Introducing a small amount of HAc, the holes in the corresponding perovskite film become much smaller and the surface coverage of perovskite films is increased (Fig. 1b). When increasing amounts of HAc is used in the precursor solution, the quality of the perovskite films is gradually improved, until few holes remain in the films (Fig. 1c,d). According to the work by Ulrich Wiesner and co-workers, formation of perovskite films could be divided into four steps: evaporation of the solvent, diffusion of the excess uncorrelated salt out of the precursor structure, removal of the uncorrelated salt from the film and removal of stoichiometric MAI from the perovskite lattice^27^. The introduction of HAc can have obvious impacts on the second and third steps based on understanding of the normal chemical process. The process is depicted by the reaction equation (1). The generated CH_3_NH_3_Ac is more volatile than CH_3_NH_3_I, which can shorten the diffusion time of the excess uncorrelated salt out of the precursor structure and removal time of the uncorrelated salt. This indicates that HAc has the ability to accelerate the formation of supersaturated perovskite solution during spin-coating process, which improves surface coverage of perovskite films^28^. In addition, HAc may enhance the solubility of PbI_2_ to increase the amounts of perovskite in precursor solution^29^.


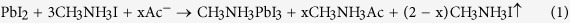



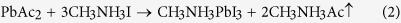


**Figure 2:**
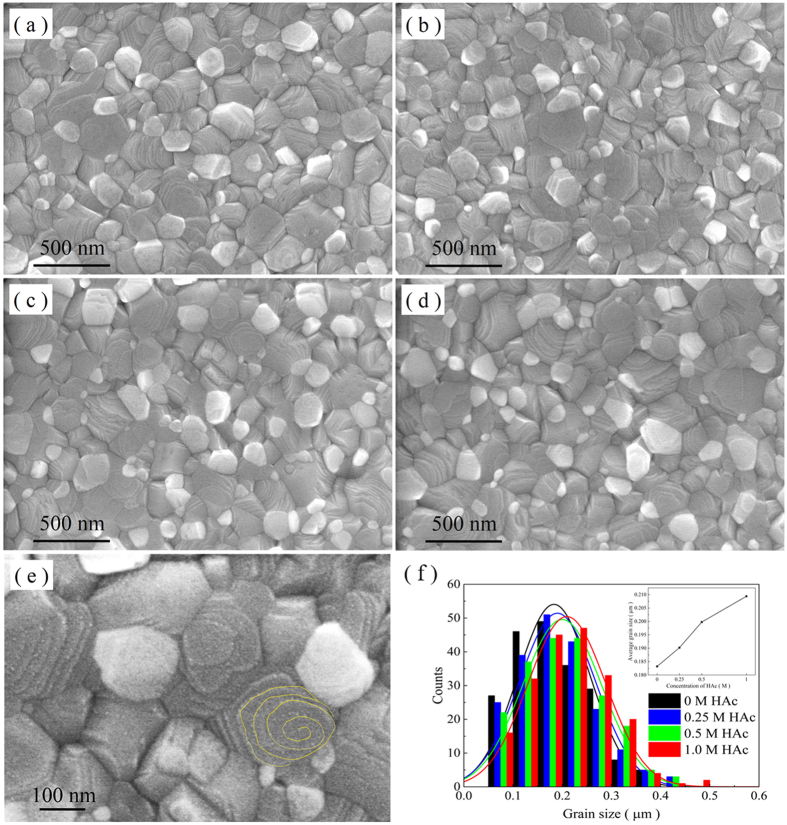
The morphology of films based on PbAc_2_ by SEM. (**a**–**d**) The image of perovskite films deposited on a FTO/compact-TiO_2_ substrate from the precursor solution containing different amounts of HAc, 0 M, 0.25 M, 0.5 M and 1.0 M, respectively. (**e**) SEM partial enlarged detail of (**c**) showing characteristic of spiral growth of the perovskite grains. (**f**) A histogram comparing the difference of perovskite grain size formed with different amounts of HAc, based on a set of data measured with 200 grains for each sample. The inset in (**f**) shows the change of average grain size with different amounts of HAc.

When PbAc_2_ is used as lead source substituting for PbI_2_, the obtained perovskite films are obviously homogenous and continual, with almost full surface coverage (Fig. 2 and Supplementary Fig. 1). The large magnification SEM images show that the perovskite films are made of high-crystallinity grains with a size of 80~300 nm (Fig. 2). It can be found that the perovskite grains present a step-like morphology. According to crystal growth theory, this should be a result generated by the spiral growth mechanism^30^. Figure 2e shows clearly the spiral growth characteristics. In the case, the chemical process can be described by the reaction equation (2). As one of the reaction products, CH_3_NH_3_Ac is instantaneously decomposed to CH_3_NH_2_ and HAc on the hot plate during the so-called spurious sublimation process, and the high-quality perovskite CH_3_NH_3_PbI_3_ is obtained on the substrate. With increasing HAc concentration in the precursor solution, vapor pressure of HAc becomes higher during spin-coating, which slows down sublimation of CH_3_NH_3_Ac. As a result, perovskite crystals have a prolonged growth period, which increases the size of some crystals as shown from the Fig. 2a to Fig. 2d. It should be noted that the time available for perovskite crystals growth is limited by solvent evaporation and CH_3_NH_3_Ac residence time in the process. The added HAc can only enlarge perovskite crystals by increasing the CH_3_NH_3_Ac residence time since introduction of HAc has no obvious impact on solvent evaporation. Generally, large crystals are beneficial for light absorption and electron transport in the photoelectrical films^31^. On the other hand, the presence of excess HAc in the precursor solution may result in pin-holes by the rapid volatilization of residual HAc in the perovskite films, as shown in Supplementary Fig. 1d.

**Figure 3:**
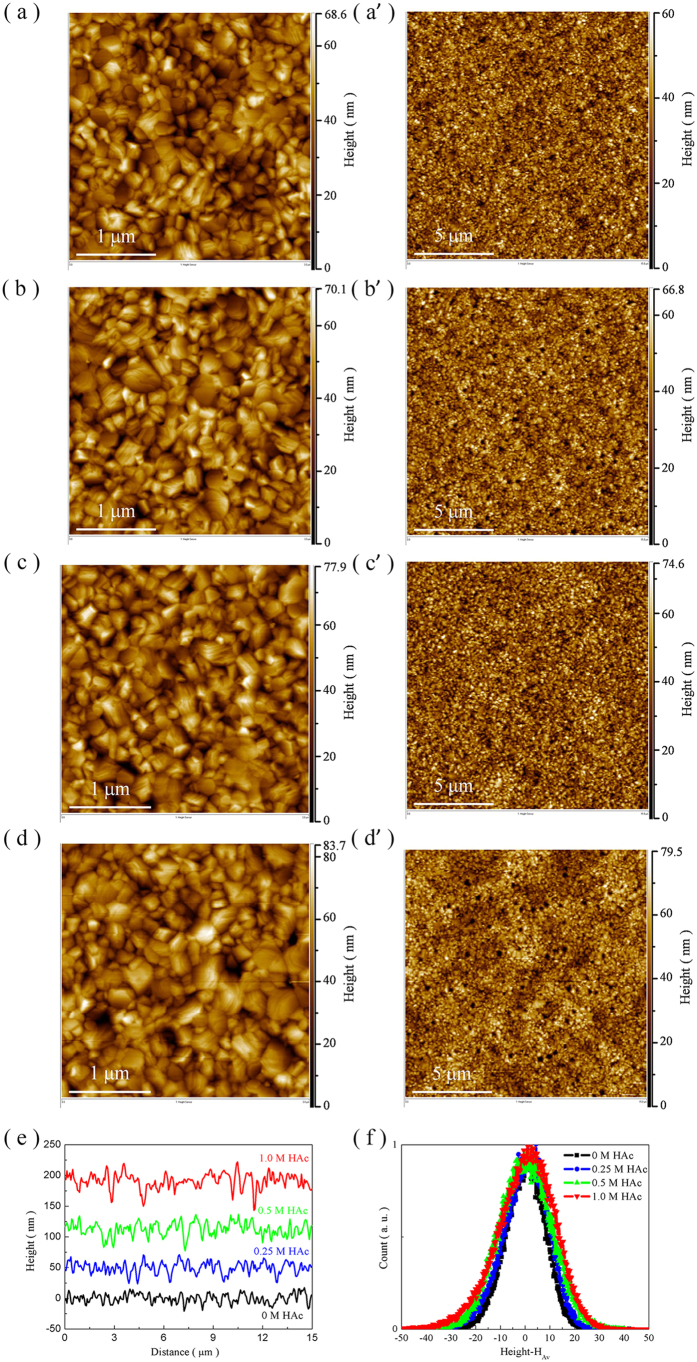
The morphology of films based on PbAc_2_ by AFM. (**a**–**d**) The image of perovskite films deposited on a FTO/compact-TiO_2_ substrate from the precursor solution containing different amounts of HAc, 0 M, 0.25 M, 0.5 M and 1.0 M, respectively. (**e**) Line segments from each scanning upon a range of 15 μm by 15 μm. (**f**) Depth distribution with around an average height, *H*_*Av*_, for each group.

The perovskite films based on PbAc_2_ with different amounts of HAc are further characterized by AFM. As shown in Fig. 3, the perovskite crystals with layered structure have a random arrangement on a FTO/compact-TiO_2_ substrate. With the increasing amounts of HAc in the precursor solution, the layered perovskite crystals have slight growth along the non-uniform orientations of crystals plane since the time of removal of CH_3_NH_3_Ac from the perovskite films is extended. This leads to the phenomena that the obtained film forms a more undulating surface after the addition of HAc. Roughness of the perovskite films is characterized by line segments (Fig. 3e) and depth distribution (Fig. 3f). The arithmetical mean deviation of the profile (*R*_*a*_), root mean square deviation of the profile (*R*_*ms*_) and the max height of the profile (*R*_*max*_) of the films are calculated and the values are presented in Supplementary Table 1. Provided that the continuous interface of perovskite/HTL is not violated, the slight increase in roughness derived from the relatively larger grains is not only beneficial for light harvesting by extending light retention time in the perovskite films, but also can improve charge transporting properties due to the enlarged contact area^32^,^33^.

To further investigate the influence of HAc on perovskite structure, XRD of the perovskite films based on PbAc_2_ with various amounts of HAc is carried out (Supplementary Fig. 2). In the XRD patterns, the peaks located at 13.9°, 20.0°, 28.3°, 31.7°, 40.7°, and 43.0° indicate a tetragonal crystal structure of the CH_3_NH_3_PbI_3_ perovskite in the 2*θ* range of 5–65° 20,34,35. Meanwhile, some of perovskite diffraction signals such as the peaks at 20.0° and 31.7° are absent or show a very low intensity, since crystals prefer to grow along the [001] direction^36^. As shown in Supplementary Fig. 2, the peak of PbI_2_ impurity appears at 12.5° in the case of 0.5 M PbAc_2_ and 1.5 M MAI which is under iodine-rich conditions. With increasing amounts of HAc, the peak intensity of the PbI_2_ impurity at 12.5° becomes obviously weaker. According to the report from H. Sargent *et al*., the formation energy of the perovskite relative to its decomposition into MAI and PbI_2_ phases has a low value of about −0.1 eV under iodine-rich conditions^37^. This indicates that PbI_2_ and MAI easily coexist with perovskite. The introduction of HAc can undoubtedly restrain the formation of PbI_2_ due to chemical equilibrium force. In the case of 1.0 M HAc, the PbI_2_ impurity peak at 12.5° almost disappeared. In brief, the introduction of HAc to the precursor solution can inhibit the decomposition of perovskite to improve purity of the films, which is advantageous in achieving high photovoltaic performance of the perovskite solar cells.

### Electronic properties of perovskite films

**Figure 4:**
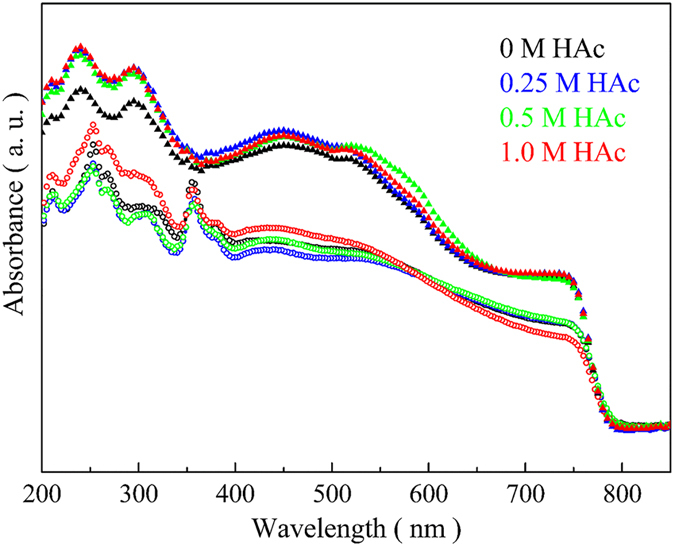
Absorption analysis by UV-vis. The spectra of perovskite films deposited on a FTO/compact-TiO_2_ substrate with different amounts of HAc. Solid triangle ▲ and open circle ○ are for PbAc_2_ and PbI_2_, respectively.

UV-visible spectra of the perovskite films prepared from the precursor solution with PbAc_2_ and various amounts of HAc are shown in Fig. 4 with the results from PbI_2_ for comparison. In order to avoid the effect of thickness on electronic properties of perovskite films, cross-sectional SEM images show that introduction of HAc have no noticeable influence on the films thickness, which is approximately 260 nm (Supplementary Fig. 3a–d). Similarly, there is few difference on thickness between PbI_2_ and PbAc_2_ used as lead source to prepare perovskite films (Supplementary Fig. 3e). All the perovskite films show absorbance in the overall visible region with threshold at about 780 nm, which is in accordance with the reported results^38^,^39^,^40^. The intensities of all peaks in the UV region for the films based on PbAc_2_ are obviously higher than those based on PbI_2_ due to the improved surface coverage and enlarged grain size as presented in the SEM results, showing a higher light adsorption ability. In UV-visible spectra, the peaks in the range from 100 nm to 300 nm are normally attributed to n → σ^*^ or π → π^*^, and the peaks located in the region higher than 300 nm represent the transition of n → π^*^^41^. Using PbI_2_ as lead source, the peaks around 210 nm and 240 nm are attributed to n → σ^*^ of CH_3_NH_3_PbI_3_. According to the work by H. Sargent, there are coordination complexes formed under iodine-rich conditions^36^. The peak at about 360 nm is attributed to n → π^*^ of these coordination complexes as impurity for the perovskite films. In the case of PbAc_2_, the peak at about 360 nm disappears, indicating a higher purity of CH_3_NH_3_PbI_3_. Evidently this is beneficial for photo-generated electron transport in the perovskite films by reducing impurity-induced trap sites. In addition, the adsorption intensity increases gradually with increasing HAc amounts when PbAc_2_ is used as lead source. Therefore, it is believed that the perovskite films derived from acetate-rich precursor solution can provide more efficient generation and transportation of electron.

**Figure 5:**
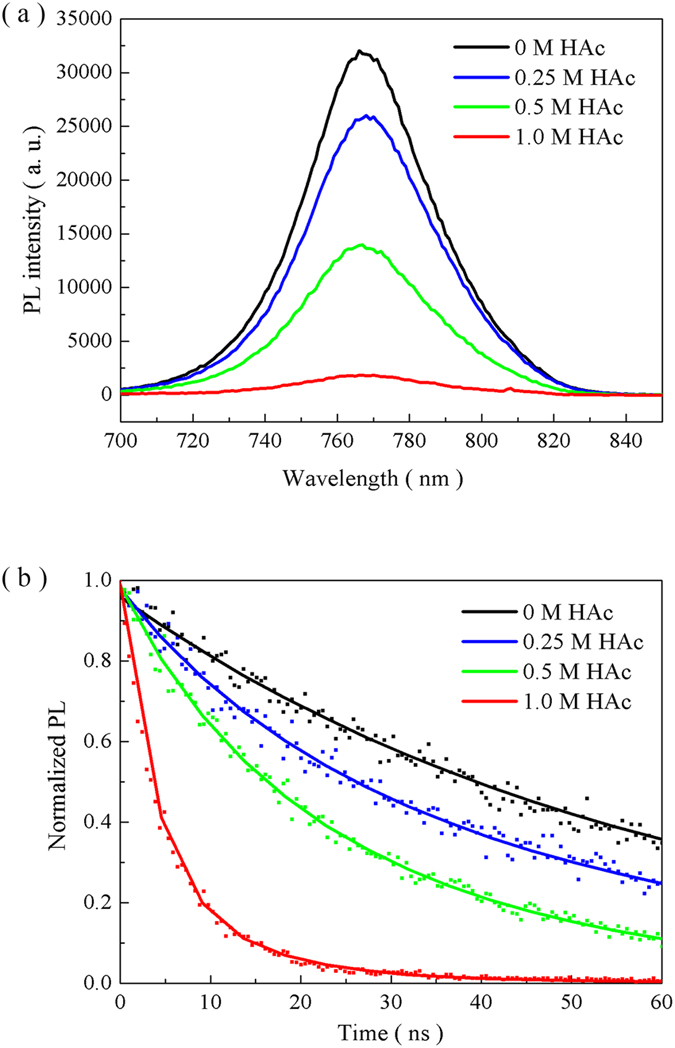
The films’ electronic analysis by PL. (**a**) Steady-state and (**b**) time-resolved spectra of perovskite films deposited from the solution with PbAc_2_ as lead source containing different amounts of HAc on a FTO/compact-TiO_2_ substrate.

To further investigate the influence of HAc on electronic properties of perovskite films, steady-state and time-resolved photoluminescence (PL) spectra of the perovskite films are measured. As shown in Fig. 5a, PL peaks appear in the range of 740–810 nm, which is consistent with the results reported before^42^,^43^. With increasing amounts of HAc in the precursor solution, the PL peak intensity of the obtained perovskite films decreases gradually. Considering the fact that the perovskite films are continual and have almost full surface coverage on the substrates, the reduced PL peak intensity can be attributed to a faster electron extraction from the perovskite films to the compact TiO_2_ layers^44^. More efficient carrier transport achieved by the faster electron extraction maybe a contribution to the enhancement in the photovoltaic efficiency^45^. The time-resolved PL spectra of perovskite films provide further an evidence for the faster electron extraction process (Fig. 5b). The fitted curves and the corresponding decay time values are acquired by fitting the dates with biexponential decay function. The decay time value is 59.7 ns for the perovskite film prepared without HAc. In the cases involving HAc, the decay time values are 16.9 ns, 10.9 ns and 3.9 ns for 0.25 M, 0. 5 M and 1.0 M HAc, respectively, indicating the faster electron extraction processes. It is believed that PbI_3_^−^, PbI_4_^2−^, and PbI_5_^3−^ are formed through the recombination of Pb^2+^ and I^−^ since the local stoichiometry in the precursor solution is violated^37^. These complex ions produce a motif similar to the Pb^0^_I_ neutral anti-site defect that can shorten charge diffusion length and inhibit effective charge separation. As HAc is added in the precursor solutions, plenty of Ac^−^ competes with I^−^ in order to combine with Pb^2+^, reducing existence of these complex ions. Therefore, introduction of HAc can reduce the Pb^0^_I_ neutral anti-site defects and achieve more effective charge separation.

### Photovoltaic performance of perovskite solar cells

**Figure 6:**
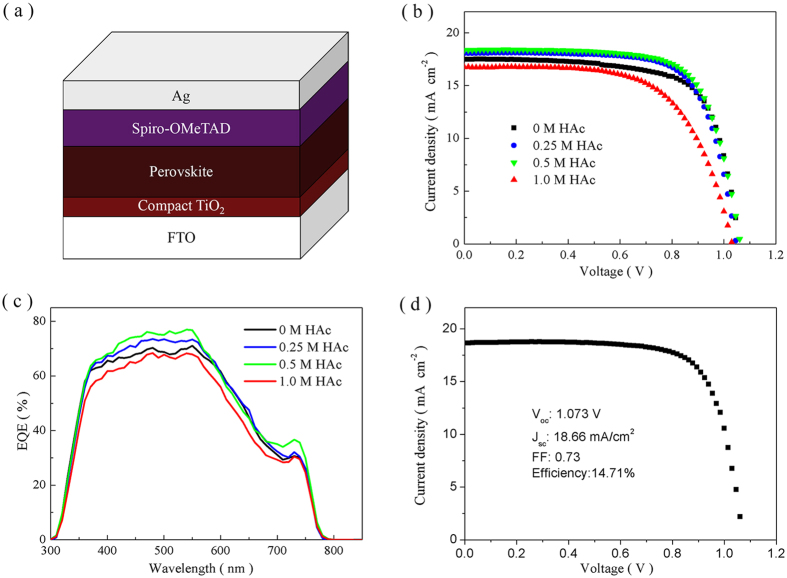
Solar cells performance. (**a**) A schematic structure of perovskite solar cells. (**b**) Typical *J-V* curves and (**c**) external quantum efficiency (EQE) spectra of the perovskite solar cells formed from the solution with PbAc_2_ as lead source containing different amounts of HAc. (**d**) *J-V* curves of the champion perovskite solar cells with 0.5 M of HAc.

The perovskite solar cells are fabricated by one-step method using a planar device configuration (Fig. 6a). Typical *J-V* curves of the same batch of devices at optimized condition are measured (Fig. 6b) and the corresponding photovoltaic parameters are shown in Table 1. Under the used fabrication conditions described in the experimental section, the photovoltaic performance of the devices using PbI_2_ as lead source is very poor because the fabrication conditions are optimized not for PdI_2_ but rather for PdAc_2_ (Supplementary Fig. 3), in accordance with the reported results^20^,^27^. However, it is clear that the photovoltaic performance can obviously be improved by introduction of HAc as shown in Supplementary Fig. 4 and Supplementary Table 2. The numerous holes in the perovskite films using PdI_2_ as lead source (Fig. 1) cause serious charge recombination, which can be avoided partially by introduction of HAc. This indicates that a full surface coverage is crucially important for a high-performance perovskite solar cell^46^,^47^. When PbAc_2_ is used as lead source, all the perovskite cells show good performance. With introducing the moderate amount of HAc, power conversion efficiency (PCE) of the corresponding solar cells is improved due to the increase of short-circuit current density (*J*_*sc*_). *R*_*sh*_ of devices with no HAc is 33761.90 ohm, which increased to 197280.59 ohm as the amount of HAc in precursor solution increased. It has been discussed that a motif similar to the Pb^0^_I_ neutral anti-site defect easily exists in perovskite films because of the violated local stoichiometry when HAc is not added in the precursor solution. The defect may increase the opportunity of charge recombination, which resulted in the decreased *R*_*sh*_. The best efficiency of devices based on 0.5 M HAc can be explained by the balance of high *R*_*sh*_ and low *R*_*s*_. The external quantum efficiency (EQE) spectra show a consistent response (Fig. 6c). This can be attributed to the increased light absorption of the perovskite films, less charge recombination and faster charge extraction, as discussed above. The device with the best photovoltaic performance is achieved from the solution involving 0.5 M of HAc. A PCE of 14.71% is obtained with *V*_*oc*_of 1.073 V, *J*_*sc*_ of 18.66 mA · cm^−2^, and *FF* of 0.73 (Fig. 6d). However, adding HAc in excess to the precursor solution causes the performance of the corresponding device to decline because of the existence of pin-holes in the perovskite film as shown in Fig. 2d. In addition, the similar conclusion can be drawn from the *J-V* curves obtained by scanning in forward and reverse bias directions despite their significant difference between reverse and forward scan (Supplementary Fig. 5 and Supplementary Table 3). As found in some works, the scanning speed and width have an influence on this difference as the hysteresis for perovskite solar cells, and high capacitance at a low frequency of perovskite and photo-induced halide ion migration play a main role in device hysteresis^10^,^48^,^49^. The issue of contact resistance may also influence the hysteresis, which can be exaggerated by recombination with opposite carriers at the heterojunctions between the perovskite and the metallic electrodes^50^. Understanding and resolving the hysteresis is crucial for further improvement in performance of perovskite solar cells.

To further corroborate the improvement, the deviation and average values of photovoltaic parameters are given in Supplementary Fig. 6. The average PCE of devices are 12.34%, 12.77%, 12.95% and 10.39% for 0 M, 0.25 M, 0.5 M 1.0 HAc in the precursor solutions, respectively. As for the devices fabricated by using the moderate amounts of HAc, the average *J*_*sc*_and *FF* are improved, and the average *V*_*oc*_at around 1.037 V has no significant changes except for the one involving 1.0 M HAc. In addition, the reproducibility of perovskite solar cells formed from precursor solution containing 1.0 M of HAc has an obvious deterioration.

## Conclusions

The influences of acetate ions on morphology, structure, electronic properties, and photovoltaic performance of perovskite films are investigated. Utilizing a suitable amount of acetate ions, a homogeneous, continual and almost perfect perovskite film can be formed and the basal crystal grains have high crystallinity and relatively large size. The perovskite films based on PbAc_2_ with HAc have higher light absorption ability, more efficient electron transport and faster electron extraction from perovskite to electron transport layer. For the planar perovskite solar cells fabricated by the simple process of one-step spin-coating and short-annealing time, the champion PCE of 14.71% is achieved. This work provides a simple but efficient approach to obtain high-quality perovskite films in order to improve photovoltaic performance of a perovskite solar cell.

## Methods

### Preparation of perovskite precursor solution

Prior to preparation of perovskite precursor solution, methylammonium iodide (MAI) was synthesized according to the procedure reported^11^. Firstly, hydroiodic acid solution (30 mL, 57 wt% aqueous solution, J&K) was gradually added dropwise to methyl amine water solution (32 mL, 40 wt% aqueous solution, J&K), then the mixture was continuously stirred in an ice-bath for 3 hours under Ar atmosphere. Secondly, the mixed solution went through rotary evaporation at 60 °C for 2 hours. The precipitated crystals were dissolved in ethanol, and recrystallized with hexane at room temperature. The procedure of recrystallization was repeated thrice. The obtained crystals were filtered and washed with diethyl ether. Finally, the resulting white powder was dried overnight in a vacuum drying oven before use.

To generate the perovskite precursor solution, MAI was blended with PbAc_2_·3H_2_O (0.5 M, J&K) or PbI_2_ (0.5 M, Sigma-Aldrich) in anhydrous N,N-dimethylformamideat a 3:1 molar ratio, respectively^20^. Subsequently, the precursor solutions containing 0.25 M, 0.5 M, 1.0 M of acetic acid were prepared by adding different amounts of acetic acid. The precursor solutions were stirred for 10 min at room temperature.

### Fabrication of perovskite solar cell

Perovskite solar cells were fabricated using FTO-coated glass as substrate with a size of 20 × 20 mm (15 Ω per square, Nippon Sheet Glass). Initially, FTO glass was etched with HCl and zinc powder to form a testing area for avoiding short circuit. Substrates were cleaned sequentially in hot ethanol, propan-2-ol and ethanol by an ultrasonic cleaner. Compact TiO_2_ as a hole-blocking layer was deposited by spin-coating a titanium dioxide sol at 4,000 r.p.m. for 30 s, and annealed at 450 °C for 60 min in a muffle furnace. The titanium dioxide sol was prepared according to the literature procedure^51^. The perovskite film was then deposited by spin-coating the perovskite precursor solution at 2,000 r.p.m for 60 s in an argon-filled glovebox. Afterwards, the films were annealed at 150 °C for 5 min. The hole-transporting layer (HTL) was generated from a solution containing 72.3 mg 2,2′,7,7′-tetrakis(NN-di-p-methoxyphenylamine)-9,9-spirobi-fluorene (spiro-MeOTAD), 17.5 μL of bis(trifluoromethylsulphonyl)imide (LiTFSI) in acetonitrile (520 mgmL^−1^), 28.8 μL 4-tert-butylpyridine (TBP), and 1 mL chlorobenzene. Finally, 65 nm of silver electrodes were thermally evaporated on the spiro-MeOTAD layer.

### Characterization

X-ray diffraction (XRD) patterns were obtained by a Rigaku MiniFlex II X-ray diffractometer. The morphology of perovskite films was investigated by scanning electron microscopy (SEM, Hitachi SU8010) and atomic force microscopy (AFM, Bruker dimension icon). The AFM images with a resolution of 256 × 256 were scanned upon a range of 3 μm by 3 μm and 15 μm by 15 μm, respectively^20^. For surface roughness, at least six samples were measured for each group and the average values were presented. The light absorbance spectra were acquired using an Agilent Cary 300 conc UV-vis spectrophotometer. Steady-state and time-resolved photoluminescence (PL) measurements were carried out using a time-resolved single-photon counting technology (Edinburgh FLS 980). The wavelength of exciting light was 507 nm with a pulse duration of 100 ps at frequencies between 1–10 MHz. Photoluminescence spectra were obtained at the wavelength range of 500 to 800 nm with 1 nm increment.

### Measurements

Current density-voltage (*J-V*) characteristic curves of perovskite solar cells were measured by a source meter (Keithley, 2420) with a solar simulator (Newport, Oriel Sol 3 A) under 100 mW·cm^−2^ AM 1.5 G illumination, standardized by a silicon reference solar cell (Oriel Instrument). Typical *J-V* curves were obtained from scanning in reverse bias direction at the step width of 100 mV. The active area of the devices was defined using a non-reflective metal aperture of 0.1 cm^2^. The voltage was scanned from 1.5 V to 0 V at a rate of 100 mV·s^−1^. The external quantum efficiency (EQE) spectra were obtained using a monochromatic incident light produced by a power source (Newport 300 W Xenon lamp, 66902) with a monochromator (Newport Cornerstone 260). The data acquisition was accomplished under DC mode with a power meter (Newport 2936-C).

## Additional Information

**How to cite this article**: Zhao, Q. *et al*. Improving the photovoltaic performance of perovskite solar cells with acetate. *Sci. Rep.* **6**, 38670; doi: 10.1038/srep38670 (2016).

**Publisher's note:** Springer Nature remains neutral with regard to jurisdictional claims in published maps and institutional affiliations.

## Supplementary Material

Supplementary Information

## Figures and Tables

**Table 1:** Photovoltaic parameters of perovskite solar cells based on PbAc_2_ as lead source with different amounts of HAc.

HAc Concentration (M)	*V*_*oc*_ (V)	*J*_*sc*_ (mA · cm^−2^)	*FF*	*PCE* (%)	*R*_*sh*_ (Ω)	*R*_*s*_ (Ω)
0	1.058	17.52	0.70	13.00	33761.90	84.13
0.25	1.047	18.07	0.71	13.48	75595.58	61.41
0.5	1.063	18.40	0.71	13.86	197280.59	67.68
1.0	1.031	16.73	0.62	10.85	122458.15	93.82

V_oc_, open circuit voltage; J_sc_, short circuit current; FF, fill factor; PCE, photoconversion; R_sh_, shunt resistance; R_s_, series resistance.
