# Supplementary material for: Improving the photovoltaic performance of perovskite solar cells with acetate
Source: Sci Rep. 2016 Dec 9;6:38670. doi: 10.1038/srep38670 (PMC5146662; doi:10.1038/srep38670)
Supplement: Supplementary Information [file srep38670-s1.pdf]

Electronic Supplementary Information (ESI) for

Improving the photovoltaic performance of perovskite solar cells with acetate

Qian Zhao [a], Guoran Li [a*], Jian Song[b], Yulong Zhao[b], Yinghuai Qiang [b*] and Xueping Gao [a]

[a]*Institute of New Energy Material Chemistry, School of Materials Science and Engineering, National Institute of Advanced Materials, Nankai University, Tianjin 300350, China. Email: guoranli@nankai.edu.cn. Tel: +86-22-2350 0780*

[b]*School of Materials Science and Engineering, China University of Mining and Technology, Xuzhou 221116, Jiangsu, China. Email: yhqiang@cumt.edu.cn.*

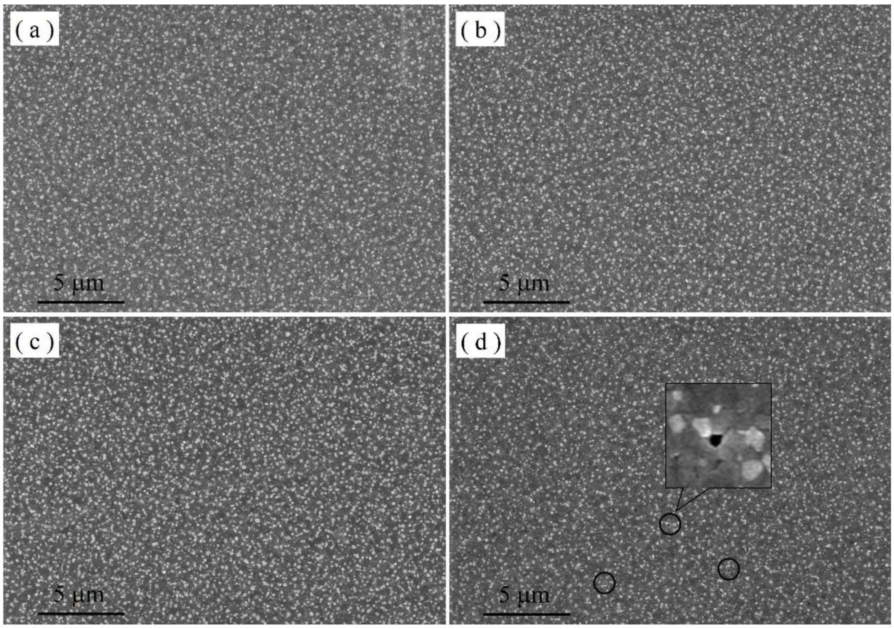

Supplementary Figure 1 | The morphology of films based on $PbAc_2$ by SEM. (a-d) SEM images of perovskite films based on $PbAc_2$ as lead source with different amounts of HAc, 0 M, 0.25 M, 0.5 M and 1.0 M, respectively. The examples of pin-holes are marked by black circles.

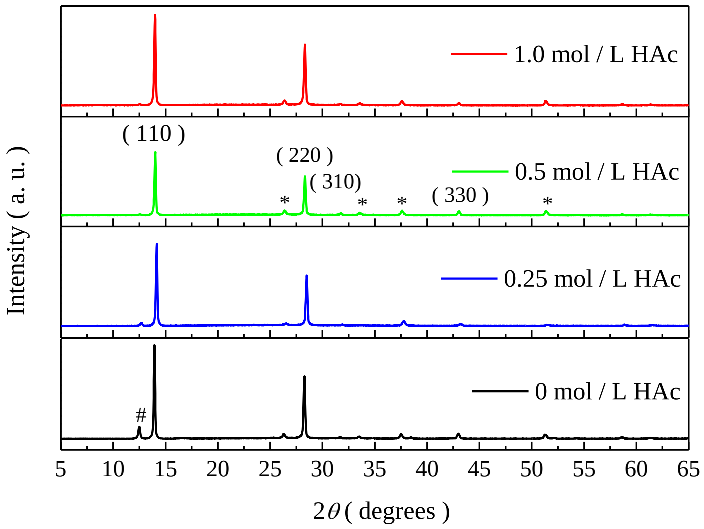

Supplementary Figure 2 | Structure analysis by X-ray diffraction measurement. XRD pattern of perovskite films based on $PbAc_2$ as lead source with different amounts of HAc. Peaks signed * and # are attributed to the FTO and PbI_2, respectively.

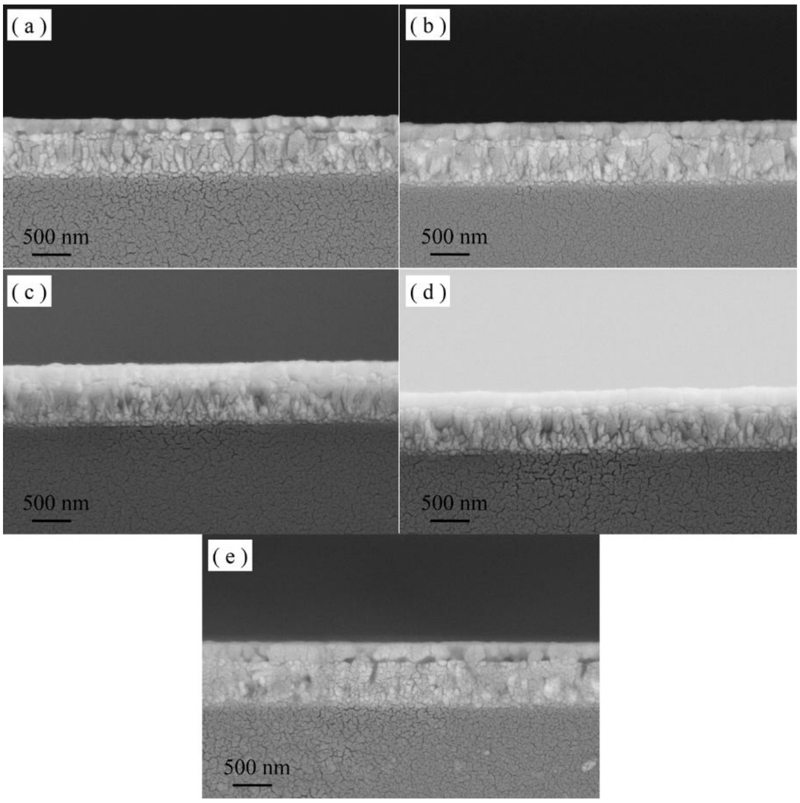

Supplementary Figure 3 | The films' thickness analysis by SEM. (**a-d**) Cross-sectional SEM images of perovskite films based on $PbAc_2$ as lead source with different amounts of HAc, 0 M, 0.25 M, 0.5 M, 1.0 M, respectively. (**e**) Cross-sectional SEM images of perovskite films based on PbI_2 as lead source without HAc.

Supplementary Table 1 | Surface roughness parameters of perovskite films based on $PbAc_2$ as lead source with different amounts of HAc.			
**Concentration of HAc (M)**	**R_a (nm)**	**R_{ms} (nm)**	**R_{max} (nm)**
0	6.77	8.48	67.95
0.25	7.47	9.34	75.33
0.5	8.52	10.68	87.43
1.0	8.53	11.02	114.44

R_a, arithmetical mean deviation; R_{ms}, root mean square deviation; R_{max}, the max height.

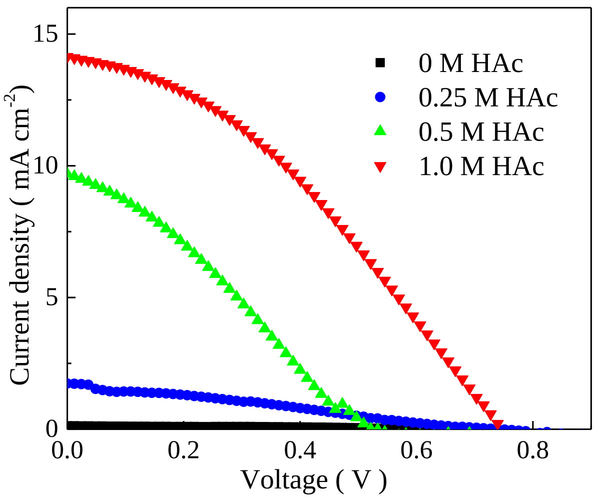

Supplementary Figure 4 | *J–V* curves of perovskite solar cells based on PbI_2 as lead source with different amounts of HAc.

Supplementary Table 2 | Photovoltaic parameters of perovskite solar cells based on PbI_2 as lead source with different amounts of HAc.				
**HAc Concentration (M)**	**V_{oc} (V)**	**J_{sc}(mA cm^{-2})**	**FF**	**PCE (%)**
0	0.587	0.13	45.45	13.00
0.25	0.733	1.73	26.38	13.48
0.5	0.535	9.69	28.88	13.86
1.0	0.745	14.15	35.81	10.85

V_{oc}, open circuit voltage; J_{sc}, short circuit current; FF, fill factor; PCE, photoconversion; R_{sh}, shunt resistance; R_s, series resistance.

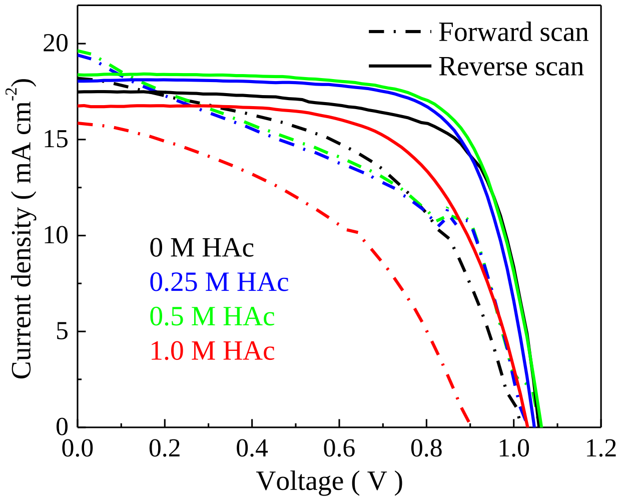

Supplementary Figure 5 | *J–V* curves of perovskite solar cells based on PbI_2 as lead source with different amounts of HAc by scanning in forward bias directions at the step width of 100 mV.

Supplementary Table 3 | Photovoltaic parameters of perovskite solar cells based on PbI_2 as lead source with different amounts of HAc by scanning in forward bias directions at the step width of 100 mV.

HAc Concentration (M)	V_{oc} (V)	J_{sc}(mA cm^{-2})	FF	PCE (%)
0 M	1.015	18.26	50.68	9.40
0.25 M	1.033	19.48	48.03	9.66
0.5 M	1.052	19.72	47.02	9.76
1.0 M	0.902	15.90	46.00	6.60

V_{oc}, open circuit voltage; J_{sc}, short circuit current; FF, fill factor; PCE, photoconversion; R_{sh}, shunt resistance; R_s, series resistance.

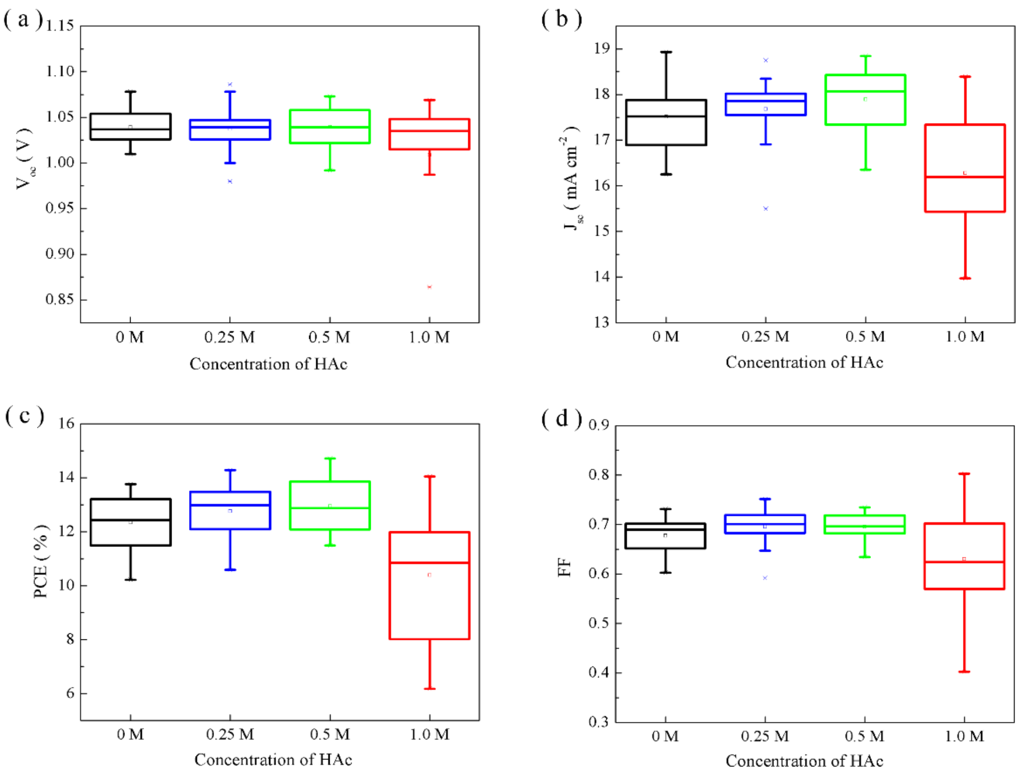

Supplementary Figure 6 | Distribution on Photovoltaic parameters pertaining to different concentration of HAc. Data from 31 cells under each condition are used for the box charts.
